# Hepatotoxicity Induced by Azole Antifungal Agents: A Review Study

**DOI:** 10.5812/ijpr-130336

**Published:** 2023-04-09

**Authors:** Amin Rakhshan, Bardia Rahmati Kamel, Ali Saffaei, Maria Tavakoli-Ardakani

**Affiliations:** 1 Department of Clinical Pharmacy, School of Pharmacy, Shahid Beheshti University of Medical Sciences, Tehran, Iran; 2 Students' Scientific Research Center, Tehran University of Medical Sciences, Tehran, Iran; 3 Pharmaceutical Sciences Research Center, Shahid Beheshti University of Medical Sciences, Tehran, Iran

**Keywords:** Liver Injury, Antifungals, Azoles, Adverse Effects, Hepatotoxicity

## Abstract

**Context:**

Fungal infections are very common, and several medications are used to treat them. Azoles are prescribed widely to treat fungal infections. In addition to therapeutic effects, any drug can be accompanied by side effects in patients. One of the most important complications in this regard is liver injury. Therefore, hepatotoxicity induced by azole antifungal drugs were reviewed in this study.

**Evidence Acquisition:**

English scientific papers were evaluated to review the effects of hepatotoxicity by azole antifungal agents, and the related studies' results were summarized using a table. The systematic search was implemented on electronic databases, including PubMed, Google Scholar, and Science Direct. Original articles and review articles that were published before April 1, 2022, were included in the study. Those articles without available full text or non-English articles were excluded. Also, articles that reported pediatric data were excluded.

**Results:**

Most studies have reported the effects of hepatotoxicity by azole antifungal agents, and their mechanisms have been described.

**Conclusions:**

Clinical evaluations regarding the hepatotoxicity of antifungal agents provided in the literature were reviewed. Therefore, it is recommended to prescribe these drugs with caution in high-risk patients suffering from liver diseases, and patients should be monitored for hepatotoxicity. However, more research is needed to evaluate the hepatotoxicity of azole antifungal agents and select appropriate drugs according to cost-effectiveness and the side effects' profiles, relying on lower incidence of this liver complication.

## 1. Context

Fungal infections in humans and animals have increased significantly in recent years in terms of severity and prevalence. Fungi are eukaryotic organisms whose resemblance to mammalian cells has limited development and provision of new drugs against them. These infections can affect people of any age (1). Pharmacological treatment of fungal diseases has evolved with the discovery of oral and relatively non-toxic azole antifungal drugs. Recently, new formulations of these drugs have become available. Antifungal agents currently available fall into several categories, namely systemic and topical antifungal agents. Systemic drugs are prescribed both orally or intravenously and used to treat systemic, mucosal, and cutaneous fungal infections. Topical antifungal agents are also prescribed for the management of mucosal and cutaneous fungal diseases but not systemic infections. Azoles are among the topical and systemic antifungal drugs introduced since the 1980s, playing an increasingly important role in managing the fungal disease. Previously, the drugs used to treat aggressive fungal disease were limited. Griseofulvin, the first introduced antifungal agent, was used to treat invasive fungal diseases, but it was indicated to be appropriate for treating dermatophytes with limitations. Although flucytosine had a broader spectrum of activity, it could develop resistance to monotherapy. Polyene antifungals, such as amphotericin deoxycholate, were used sparingly due to their aggressive method of administration (intravenously) and high side effects. As a result, over the past 35 years, the use of drugs from the azole class has expanded a lot from older imidazoles, such as ketoconazole, miconazole, and clotrimazole, with limited therapeutic effects for the management of superficial mycoses to newer azoles (2, 3).

With the advent of azoles, first, miconazole and ketoconazole and then fluconazole and itraconazole were suggested as therapeutic strategies against invasive infections. Recently, new antifungal agents, including new azoles (voriconazole, isavuconazole, and posaconazole), liposomal amphotericin B, and echinocandins have shown to be efficient as a pharmacological treatment choice in order to treat these infections. Although, the administration of antifungal drugs may lead to treatment failure in many cases (4). In this review study, we intend to explain the degree of liver injury caused by azoles, explicate the differences between the damages caused by them, and finally suggest ways to reach comprehensive clinical judgments in dealing with a liver injury caused by the use of azoles.

For nearly two decades, azole has been used against various types of fungal infections. Azoles themselves are categorized into two distinct types, including triazoles and imidazoles. clotrimazole, miconazole, and ketoconazole belong to imidazoles. Fluconazole, isavuconazole itraconazole, posaconazole, terconazole, voriconazole are triazoles (5). Azoles are compounds inhibiting P-450-dependent 14 alpha-sterol demethylase and have high drug interactions due to the inhibition of P-450 CYP/, causing a high degree of drug-drug interactions. There are other mechanisms of action for these drugs; for example, it is said that these drugs can inhibit cellular respiration by binding to the membrane, alter membrane permeability, and can cause fungal cell death through toxic reactions with phospholipids in the fungal cell membrane (6). Imidazoles come in various topical forms, like shampoos, solutions, lotions, candies, vaginal tablets, and creams. Ketoconazole, the first oral azole choice in the market for the pharmacological management of fungal infections, is effective in treating a wide variety of candida infections. Patients receiving ketoconazole should take their pills with an acidic drink to better absorb the drug (6, 7). Among triazoles, fluconazole, the first-generation antifungal agent from triazoles, has several additional benefits over other azoles. One of these additional benefits is a broader spectrum of antifungal activity. Fluconazole is effective to treat several candida and Cryptococcus diseases (6-8). Itraconazole, like fluconazole, can be administered orally or intravenously. Its activity spectrum is similar to fluconazole, except that it is also effective on *Aspergillus* spp. Voriconazole has been approved by the FDA in 2002. This agent also has a wide spectrum of antifungal activity and is effective for treatment of *Aspergillus*, especially amphotericin B-resistant *Aspergillus* (6, 7). An acidic medium is not required to optimize absorption level of voriconazole. In addition, it has higher bioavailability than ketoconazole and itraconazole. Voriconazole should be taken one hour before or one or two hours after a meal since fatty foods reduce their absorption. Some patients who receive voriconazole experience transient visual impairments, such as photophobia, blurred vision, and changes in color vision. These side effects are not related to the administration route of this drug (orally or intravenously) (6). Posaconazole, as the latest triazole antifungal agent, was approved in 2006, and has a broad spectrum of antifungal activity; for instance, against previous azole-resistant candida species. Unlike voriconazole, posaconazole is also effective against zygomycetes. Posaconazole dosage forms are including oral suspension, tablet, and intravenous dosage form, oral suspension of which has poor bioavailability. If this drug is taken orally with fatty foods, its bioavailability will increase up to 400% (4, 9). The antifungal activity of azole agents results from the reduced ergosterol synthesis in the fungal membrane. The specificity of azole drugs is due to the fact that these drugs are more susceptible to fungal infection than human cytochrome p450 enzymes. However, like other medicines, azoles can cause side effects in some patients (6, 9). Ketoconazole was the first oral azole used clinically. It is more likely to inhibit mammalian cytochrome p450 enzymes than newer drugs. In other words, it is less selective for fungal p450 than new azoles (10). This phenomenon has two consequences. First, ketoconazole inhibits cytochrome p450 enzymes, inhibiting the synthesis of steroid hormones and causing significant endocrine changes such as infertility and menstrual disorders. Secondly, other drugs' metabolism alters, increasing their toxicity. The side effects of ketoconazole are mainly dose-dependent. Its side effects include headache, dizziness, pruritus, nausea, vomiting, abdominal pain, diarrhea, constipation, bloating, increased liver enzymes, hepatotoxicity, gynecomastia, or breast enlargement in men (6, 11).

Drug-induced hepatotoxicity or drug-induced liver injury (DILI) is a significant cause of liver disease, and one of the most important aspects is evaluating this reaction properly. DILI is relatively uncommon, but over-the-counter medicines, herbal preparations, or supplements are among the underlying factors (12). Medication-induced livers' side effects can be predictable or unpredictable, which, unfortunately, are often unpredictable. Drugs, such as paracetamol, can cause predictable liver damage in a short time (usually within a few days) (13). Medications causing unpredictable liver damage have an average delay period of 1 week to 2 months (such as phenytoin) or a long delay period of 1 year (such as isoniazid) (14, 15). These reactions occur in 1/1,000 - 1/10,000 patients taking therapeutic doses of various drugs of this class (16, 17). Factors leading to DILI include the chemical properties of the drug, environmental factors, such as concomitant use of the drug with alcohol, age, gender, underlying diseases, such as diabetes, and genetic factors (18). The incidence of DILI seems to be increasing among the general population (19). In connection with the hepatotoxicity of drugs, important points should be considered in evaluating these conditions, including hepatic injury pattern, time to onset of symptoms, the presence or absence of hypersensitivity, and toxic reaction after drug discontinuation (19, 20). It most often occurs in people who are genetically predisposed to the disease. If the drug metabolism and excretion are altered, it can lead to cellular events, such as the formation of oxidative stress, necrosis, apoptosis, haptenization, and activation of immune response (19, 21). Direct hepatotoxicity and immune system adverse reactions appear to play a major role in DILI mechanism (17). Drug metabolites can be electrophilic chemicals or free radicals subjected to various chemical reactions (22). These reactive metabolites can interact with proteins, lipids, and nucleic acids and lead to protein dysfunction, lipid peroxidation, DNA damage, and oxidative stress. They can also cause loss of energy production by directly influencing mitochondrial function. Eventually, abnormalities in cell function will lead to cell death and possible liver failure. Innate immune cells, such as natural killer (NK) cells, Kupffer cells (KC), dendritic cells (DCs), natural killer T (NKT) cells, and neutrophils play a crucial role in maintaining liver homeostasis by inducing immunogenic and tolerant immune responses (17). Liver damage by activating signals activates cells, such as the innate immune system, KC, NK, and NKT cells. These cells promote the development of liver damage by producing pro-inflammatory mediators and secretion of chemokines (23).

DILI also leads to the production of some inflammatory cytokines, involving IFN-γ, TNF-α, and IL-1β, playing a key role in causing tissue damage. In return, anti-inflammatory cytokines, including IL-10, IL-6, and IL-13, play a protective role and prevent liver damage. It should be noted that the balance between pro-inflammatory and anti-inflammatory cytokines determines the sensitivity and severity of liver damage (24). Some infections, such as HIV and hepatitis B and C, and influenza, can also influence the severity of DILI by targeting specific cytokines (24, 25). It has also been observed that humoral immune responses, mainly mediated by antibodies, cause hepatotoxicity. Although the role of humoral immunity in idiosyncratic drug-induced liver injury (iDILI) has not yet been fully elucidated, it has been shown that antidrug antibodies (ADAs) and autoantibodies detected in the serum of patients with iDILI cause significant cytotoxicity in liver cells (26).

Clinical and pathological patterns of hepatotoxicity consist of fulminant hepatitis, acute and chronic hepatitis, ductopenia, cholestasis, steatosis (steatohepatitis, macrovesicular, or microvesicular steatosis), and granulomatous hepatitis (27). Drugs cause liver damage in both dose-dependent and intrinsic DILI and non-dose-dependent or iDILI. Evidence shows that non-steroidal anti-inflammatory drugs (NSAIDs) and antibiotics, including amoxicillin-clavulanate, flucloxacillin, diclofenac, and isoniazid, causes DILI (28). More than 50% of cases of acute liver failure are secondary to hepatotoxicity by drugs, of which acetaminophen-induced hepatotoxicity is the most common type of hepatotoxicity caused by acetaminophen. Mortality of patients with acute secondary liver failure caused by drugs is common in non-acetaminophen-induced hepatotoxicity. Drug-induced liver injury results in need for liver transplantation in about 10% of cases. Incidence of jaundice with elevated transaminases in a patient with drug-induced hepatotoxicity is associated with a 10% increase in mortality rate. It should always be noted that any drugs or chemicals may lead to hepatic dysfunction. Therefore, obtaining an accurate medication history is crucial in evaluating patients with liver damage in hepatocellular or cholestasis manifestations (29-31).

All azoles are associated with hepatotoxicity. However, their toxic mechanisms are poorly understood. Hepatotoxicity caused by ketoconazole has been best characterized in experimental animals and human models. As they are a similar class of drug, details about the hepatotoxic mechanisms of ketoconazole can be used as a guide to investigate similar mechanisms of another azoles-induced hepatotoxicity (32). According to what is mentioned in various studies, the exposure of humans to triazole pesticides in various ways can lead to damage, such as neurological disorders and damage to the immune system and endocrine glands. Triazoles indicate a wide range of toxicological properties in humans and animals (33, 34). Triazole inhibits the fungal enzyme cytochrome P450 (CYP). Cytochrome P450, the major name in the large family of hemoproteins (iron-containing proteins), is often found in high concentrations in the smooth endoplasmic reticulum of liver cells (35). A significant part of fungicides' toxicity in animals occurs regarding the inhibition of CYP enzymes (36). Triazole affects the expression of several CYP genes in the liver, including multiple isoforms, CYP51, Cyp2c, and Cyp3a, xenobiotic-metabolizing enzyme (XME), and carrier genes (35, 37). It inhibits the activity of cytochrome P51 (CYP51). This cytochrome is involved in converting lanosterol to ergosterol in fungi and yeasts (34, 38). In fact, triazoles inhibit the synthesis of fungal ergosterol, resulting in a decrease in the essential sterol of the fungal cell membrane, which leads to endocrine disorders and interference with the biosynthesis of steroid hormones in mammals (33, 39, 40).

Triazoles regulate several target constitutive androstane receptor (CAR) genes. CAR plays an important role in modulating energy homeostasis, drug metabolism, and cancer development by regulating the transcription of multiple genes (41). Studies on the liver tissue show that triazoles cause to activate CAR and pregnane x receptor (PXR), induce CYP, and oxidative stress, impair cholesterol biosynthesis and alter cell signaling, and cause cell growth, cell proliferation, single cell necrosis, fat vacuolation, and apoptosis. Studies showed that triazole results in liver hypertrophy and weight gain; in the long term, its toxicity leads to liver tumors (42-44). On the other hand, triazoles can cause drug interactions in the gastrointestinal tract, liver, and kidneys, leading to tissue damage. Fifty-seven types of CYP genes were identified in the human genome, 15 of which are involved in drug metabolism (45). Among various CYPs, CYP3A4, 2C19, and 2C9 modify triazole biodegradation. CYP3A4 accounts for 30 to 60% of total hepatic CYP (46). By inhibiting the hepatic CYP, triazole hinders the biotransformation of other drugs, which can produce clinically relevant interactions. Among various drug groups with which the triazoles interact, the most important clinical interactions which prevent other drugs from deforming include Immunosuppressants, statins, anxiolytics, warfarin, antiretroviral, and benzodiazepines. Some interactions cause significant toxicity and severe liver damage (45, 47). There is very little information about the exact metabolism of triazole effect on liver tissue, and more studies are expected in the future.

The incidence of hepatotoxicity of these drugs depends on various underlying factors, involving the presence of pre-existing liver disease, genetic factors, taking concomitant hepatotoxic medications, azole dosage and plasma concentrations of drugs, and infectious liver damages caused by fungal pathogens (48, 49). Hepatotoxicity caused by itraconazole, flucytosine, and terbinafine is more common than amphotericin B-induced hepatotoxicity. The most common antifungal drug causing liver injury is ketoconazole. Hepatotoxicity induced by antifungal drugs is usually resolved spontaneously after discontinuation of the drug (17, 19). Azole antifungals have been found to be associated with DILI; international reporting databases of drugs' and adverse events from 2011 to 2014 have reported them accounting for 2.9% of all DILI (including acute liver failure events) cases (50-52). Azoles-induced hepatotoxicity can develop at any time after their administration, but many studies have demonstrated that this event usually occurs in the first month of therapy by azoles. Laboratory and clinical changes return to normal conditions after the discontinuation of these drugs. However, some cases of fulminant liver damage with or without hepatic necrosis have been seen (53). Even considering structural resemblances, limited cases of cross-reactivity between azoles have been described (54, 55). In some cases of hepatotoxicity caused by azoles, if laboratory or clinical parameters do not indicate their discontinuation, it is recommended to keep up taking the drug with continuous monitoring of liver function and plasma concentration (56). Azoles-induced hepatotoxicity features and main points about them are summarized in Table 1 (48-50, 53, 57-61).

**Table 1.** Comparison of Azoles-Induced Hepatotoxicity

Azole Drug	Hepatic Injury Pattern	Approximate Incidence of Elevations in Liver Function Tests (LFTs) (%)	Toxicity Requiring Discontinuation of the Drug	Comments
**Fluconazole**	Cholestatic	1 – 10%	elevations in LFTs that are serious enough to warrant discontinuation of the drug appeared in 0.7% of patients	Most elevations in LFTs are transient and are resolved upon drug discontinuation. There are mixed data regarding the dose-dependency of hepatotoxicity.
**Itraconazole**	Cholestatic	1 – 17.4%	1.5% of patients experience elevations in LFTs that are serious enough to warrant drug discontinuation.	Elevations in LFTs may appear between 4 – 10 weeks. The hepatocellular model of toxicity may imply severe toxicity. The dose or duration dependence of itraconazole-induced hepatotoxicity is unclear.
**Ketoconazole**	Hepatocellular	3 – 17.5%	1 in 1,000 – 3,000 patients experiences elevations in LFTs severe enough to warrant drug discontinuation.	Most LFTs elevations are transient and resolved upon drug discontinuation, but severe hepatotoxicity risk seems to be the highest among azoles.
**Posaconazole**	Hepatocellular	1 – 10%	Elevations in LFTs that are rarely severe warrant discontinuation of the drug.	Elevations in LFTs are generally resolved within two weeks after drug discontinuation.
**Voriconazole**	Mixed, hepatocellular, and cholestatic	12 – 19%	The incidence of fulminant hepatic failure is rare.	Usually, within the first 10 – 28 days of therapy, toxicity appears and may be related to the concentration of the drug.

## 2. Evidence Acquisition

**Figure 1.**
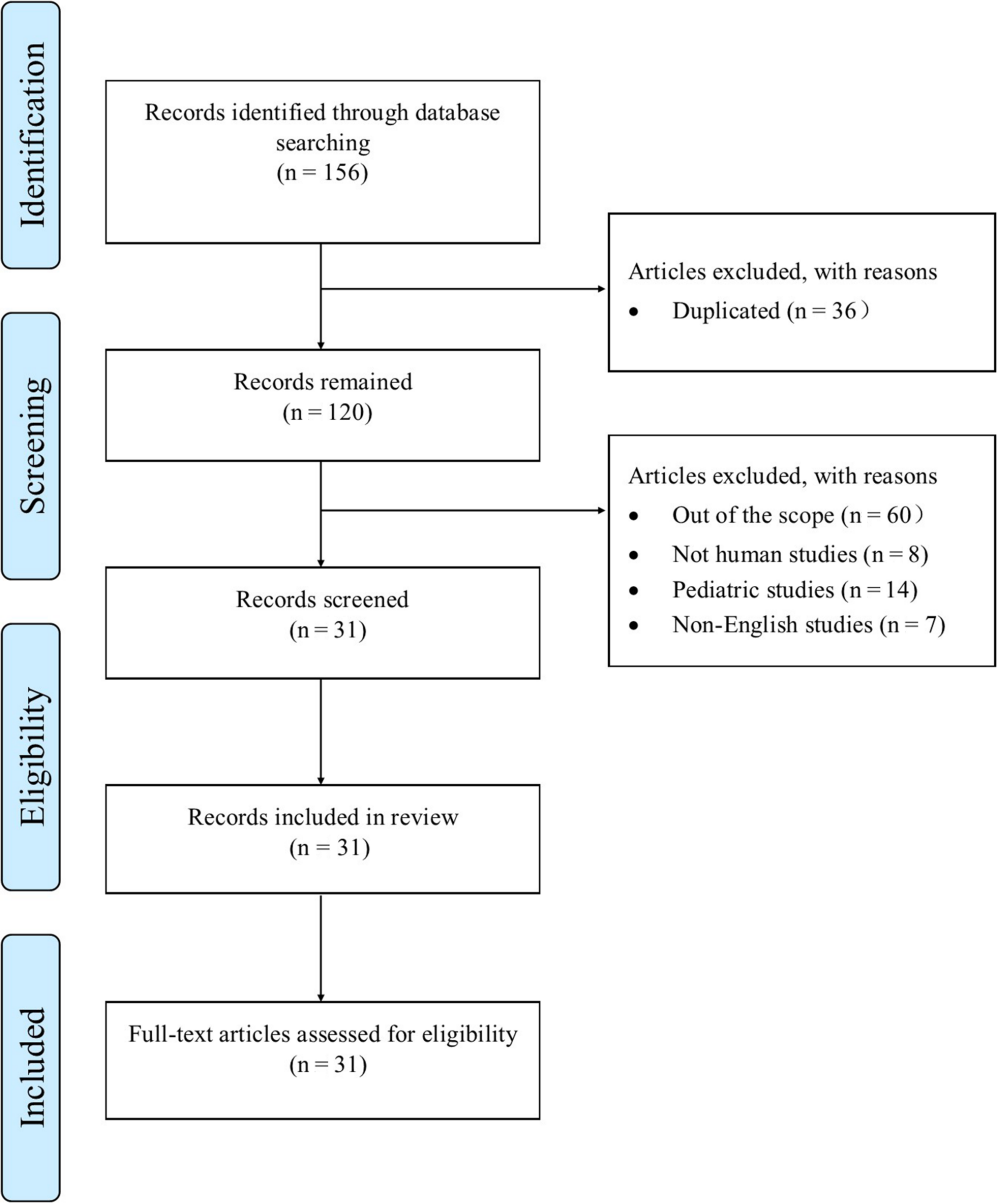
Flow diagram for search methodology

The literature search was performed on PubMed, Google Scholar, Scopus, Embase, and Science Direct databases. Original articles and review articles that were published before April 1, 2022, were included in the study. Those articles without available full text or non-English articles were excluded. Also, articles that reported pediatric data were excluded. The terms used for the literature search were "drug-induced liver injury", "antifungal agents", "azoles", and "azoles adverse effects". In the initial findings, 156 papers were evaluated. Duplicates (n = 36) and publications far from the search's objective were discarded (n = 89). Finally, 31 studies were used to write this review. A schematic summary of what has been done is shown in Figure 1.

## 3. Results

Clinical evaluations regarding hepatotoxicity of antifungal agents provided in large prospective and controlled registration trials, prospective and retrospective noncomparative cohort studies, case descriptions, and phase IV pharmacovigilance studies were reviewed. Although antifungal triazoles have shown validated potential for liver damage, experimental and clinical data have shown that the overall incidence of severe hepatotoxicity is extremely low. Among them, this review tried to emphasize the more comprehensive cases, and their level of evidence is of higher value. Results of evaluating pieces of evidence on hepatotoxicity of azole antifungal drugs are given in Table 2.

**Table 2.** Evaluation of Evidence on Hepatotoxicity of Azole Antifungal Drugs

Authors	Year of Publication	Results
**Collazos et al.**	1995	Fluconazole and itraconazole are considered safe drugs that are occasionally associated with changes in liver function and usually do not require discontinuation. Their mechanism of toxicity is unclear, although a unique reaction appears to be involved. In this study, two cases of hepatotoxicity due to fluconazole and itraconazole were reported, in whom azole discontinuation was required, and after discontinuation of the drug, symptoms were resolved immediately (62).
**Rodriguez and Acosta**	1996	The results showed that ketoconazole and miconazole caused a dose-dependent inhibition of NADH oxidase and succinate dehydrogenase, in which ketoconazole was the strongest inhibitor. Fluconazole had minimal inhibitory effects on NADH oxidase and succinate dehydrogenase. In summary, ketoconazole is a stronger mitochondrial inhibitor than the studied azoles (63).
**Adriaenssens et al.**	2001	In all three presented patients, a biochemical-histological pattern of cholestatic liver damage with interstitial bile duct damage was developed. Initial ductopenia was present in two cases, suggesting that itraconazole may be responsible for developing long-term drug-induced cholangiopathy. Jaundice was observed in all three patients. However, it was not associated with clinical signs of hypersensitivity, mostly of cellular immunoallergic and metabolic types. Itraconazole-induced liver damage is manifested with a pattern of cholestatic damage and damage to interlobular bile ducts, possibly leading to ductopenia. Thus, it is suggested to add itraconazole to the list of drugs that may be responsible for the drug-induced biliary syndrome. Further histological evidence is needed in other cases to reinforce the current findings (64).
**Somchit et al.**	2004	A statistically significant and dose-dependent increase in plasma levels of alanine aminotransferase (ALT) and alkaline phosphatase (ALP), along with dose-dependent hepatocellular necrosis, bile duct hyperplasia, and biliary cirrhosis, was found in the chronic itraconazole-treated group. Fluconazole-treated mice experienced no significant increase in levels of transaminases, but in chronic fluconazole-treated mice, only slight changes in centrilobular hepatocyte degeneration were observed. These results indicated that itraconazole caused more potent hepatotoxicity than fluconazole in rats (65).
**Wingard and Leather**	2005	The results indicated that hepatotoxicity might have a stronger association with liposomal amphotericin B than fluconazole, and the cumulative amount of the administered liposomal amphotericin B is associated with the possibility of hepatotoxicity. This is not the case with fluconazole or amphotericin B deoxycholate. These findings may be new to many, while they are clinically significant and can lead to different ways of thinking about antifungal treatment options (66).
**Somchit et al.**	2006	No changes were observed in fluconazole-treated rats. Pretreatment with SKF 525A (a non-selective inhibitor of cytochrome P450 enzymes) caused more severe hepatotoxicity in both itraconazole and fluconazole-treated rats. Dose-dependent CYP 3A activity was inhibited by itraconazole treatment. Fluconazole potently inhibited all three CYP isoenzymes. It was found that PB was involved in protecting the liver against hepatotoxicity caused by itraconazole but not fluconazole. SKF 525A increased the hepatotoxicity of both antifungal drugs in-vivo (67).
**Levin et al.**	2007	Mean serum bilirubin and levels of other liver enzymes were increased during treatment with voriconazole. There was no statistically significant difference in the maximum amount or maximum increase in liver enzymes or standard toxicity criteria (CTC)-score in relation to polymorphism of cytochrome P450. No significant relationship was observed between CYP2C9, CYP2C19, or CYP3A5 polymorphisms and serum levels of the liver enzyme in patients treated with voriconazole (68).
**Matsumoto et al.**	2009	The results showed that hepatotoxicity induced by voriconazole was observed in 34.5% of Japanese patients. It was also found that the limited doses induced hepatotoxicity because the dose was reduced in 6 patients, and the drug was discontinued in 4 patients. It was also revealed that plasma concentrations of voriconazole were a significant predictor of hepatotoxicity and that the probability of hepatotoxicity at voriconazole concentrations of 2 and 4 mg / L was equal to 1.6 and 21.6%, respectively. After the initial dose, the therapeutic range of voriconazole concentration should be 2-4 mg / l. In addition, non-linear pharmacokinetics should be considered when increasing or decreasing the dose of voriconazole. Recommended initial doses and adjustment of the next dose for the target concentration range by drug monitoring can prevent the side effects and pave the way for continuing effective treatment by voriconazole (69).
**Cadena et al.**	2009	Primary findings showed that voriconazole and inhaled amphotericin B-treated lung transplant recipients had a significantly higher hepatotoxicity risk compared to those treated with itraconazole. This hepatotoxicity was observed in 12 patients receiving voriconazole and amphotericin B. This study showed that the incidence of hepatotoxicity with prophylactic voriconazole after lung transplantation was higher than with hepatotoxicity of itraconazole. The previous trials have suggested that there is a correlation between voriconazole levels and hepatotoxicity such that, for each 1 mg/L increase in the voriconazole level, there was a 13, 7, 16, and 17% increase in levels of AST, ALT, alkaline phosphatase, and total bilirubin, respectively (70).
**Solís-Muñoz et al.**	2013	Sixty-nine percent of patients treated with voriconazole showed changes in LFTs during treatment. The increased levels of transaminases, cholestasis and a combination of both were observed in 35, 15, and 45% of cases, respectively. According to CTC classification, all patients with severe hepatotoxicity had a severe reaction. There was a correlation between the initial dose greater than 300 mg (4.5 mg/kg) and the risk of hepatotoxicity. In the control group, only 10.3% of patients experienced changes in LFTs. Therefore, voriconazole should be used cautiously in patients with severe hepatic impairment following liver transplantation through repeated monitoring of LFTs or the use of liposomal amphotericin B (71).
**Tverdek et al.**	2016	The incidence of hepatotoxicity due to antifungal therapy is widespread and is more common in patients treated with azole antifungal agents (48).
**Lo Re et al.**	2016	The incidence of the side effects in treatment with fluconazole, ketoconazole, itraconazole, and voriconazole was higher. Severe acute liver injury was uncommon following fluconazole, ketoconazole, and itraconazole consumption, while it was more common after voriconazole and posaconazole administration. One patient developed acute liver failure due to ketoconazole consumption. Pre-existing chronic liver disease increases the risk of acute liver damage (58).
**Haegler et al.**	2017	Voriconazole and fluconazole were not cytotoxic. In mitochondria isolated from rats҆ liver, ketoconazole disrupted membrane potential and cellular activity, while other azoles were not toxic. Both posaconazole and ketoconazole (but not fluconazole or voriconazole) reduced mitochondrial membrane potential in HepG2 cells exposed to the drug for 24 hours, disrupting the enzymatic function of the electron transport chain. Therefore, it can be concluded that ketoconazole and posaconazole have mitochondrial toxicity (72).
**Khoza et al.**	2017	After 14 days, only ketoconazole raised ALT and AST levels significantly higher than the control group. After 28 days, ALT levels were higher in ketoconazole, itraconazole, fluconazole, griseofulvin, and terbinafine-treated mice, respectively. AST levels were higher in mice treated with ketoconazole, followed by itraconazole, fluconazole, terbinafine, and griseofulvin, respectively. All drugs significantly increased ALP levels after 14 and 28 days of treatment. Liver enzyme levels indicated that ketoconazole had the highest risk of developing liver damage. Itraconazole, fluconazole, terbinafine, and griseofulvin were then the most effective drugs. However, histopathological changes showed that fluconazole had the highest hepatotoxicity, followed by ketoconazole, itraconazole, terbinafine, and griseofulvin, respectively (73).
**Bühler et al.**	2019	Although therapeutic drug monitoring (TDM) of antifungal agents is important in a wide range of their use in clinical settings and helps reduce the risk of liver damage, iDILI caused by antifungal azoles is a rare adverse hepatic reaction occurring at doses below the therapeutic level. The results showed cross-toxicity, dose dependence, and possible genetic predisposition to triazole-induced liver damage (74).
**Adis Medical Writers**	2020	Long-term use of systemic azole antifungals (e.g., fluconazole, isavuconazole, itraconazole, ketoconazole, posaconazole, and voriconazole) can lead to adverse drug effects, such as hepatotoxicity and cardiac adverse effects. Inhibition of cytochrome P450 by azoles can cause complications, such as adrenal insufficiency due to hormone dysfunction, while pharmacokinetic interactions can lead to myositis or peripheral neuropathy. Other notable complications include pancreatitis, bone pain, kidney failure, skin cancer, and Stevens-Johnson syndrome. Side effects can be managed by monitoring, adjusting doses, and changing azoles (75).
**Bernardo et al.**	2020	There was no difference in the incidence of hepatotoxicity in patients who achieved optimal drug plasma levels compared to those whose drug plasma levels were lower than therapeutic levels. Since, there is always the possibility of liver damage due to the use of other drugs or other accompanying causes, in this study, it was not possible to accurately attribute liver damage to posaconazole consumption; however, in other studies, the possibility of its occurrence has been rarely confirmed (76).
**Gomathi et al.**	2021	Increased hepatic levels of aminotransferases were observed after long-term use of fluconazole (more than five months with indications such as onychomycosis and dermatophytosis), and levels of liver enzymes were not increased significantly in patients taking the drug for less than 12 weeks. Therefore, in patients who receive fluconazole for a long time, it is recommended to monitor liver function markers periodically (77).

DILI occurs in 1/1,000 - 1/10,000 patients taking therapeutic doses of various drugs, including azoles. Triazole inhibits the fungal enzyme cytochrome P450 (CYP). Triazoles indicate a wide range of toxicological properties in humans and animals. Studies on the liver tissue show that triazoles cause to activate CAR and pregnane x receptor (PXR), induce CYP, and oxidative stress, impair cholesterol biosynthesis and alter cell signaling, and cause cell growth, cell proliferation, single cell necrosis, fat vacuolation, and apoptosis. Studies showed that triazole results in liver hypertrophy and weight gain; in the long term, its toxicity leads to liver tumors. Also, triazoles can cause drug interactions in the gastrointestinal tract, liver, and kidneys, leading to tissue damage. Most studies have reported the effects of hepatotoxicity caused by azole antifungal drugs, and its mechanisms have been described. The most common antifungal drug causing liver injury is ketoconazole. About itraconazole, the hepatocellular model may imply serious effects. For fluconazole, most LFTs elevations are transient and resolved upon drug discontinuation. There are mixed data regarding the dose-dependency of fluconazole-induced hepatotoxicity. Generally, for voriconazole therapy, toxicity occurs within three weeks and may be related to drug concentration. For posaconazole, severe liver damage requiring drug discontinuation is usually rare, and elevations in LFTs are generally resolved within 14 days after drug discontinuation.

## 4. Conclusions

Fungal infections have increased significantly in recent years in severity and prevalence. Pharmacological treatment of fungal diseases has evolved with the discovery of oral and relatively non-toxic azole drugs. The incidence of hepatotoxicity of these drugs depends on various underlying factors, involving the presence of pre-existing liver disease, genetic factors, taking concomitant hepatotoxic medications, azole dosage and plasma concentrations of drugs, and infectious liver damages caused by fungal pathogens. Azoles-induced hepatotoxicity is usually resolved spontaneously after discontinuation of the drug. Although antifungal triazoles have shown validated potential for liver damage, experimental and clinical data have shown that the overall incidence of severe hepatotoxicity is extremely low. However, it is recommended to prescribe these drugs with caution in high-risk patients suffering from liver diseases, and patients should be followed for hepatotoxicity. In some patients with azoles-induced hepatotoxicity, if laboratory or clinical parameters do not indicate discontinuation of them (If one of these conditions - the criteria for discontinuation of the drug following the possibility of DILI as recommended by the FDA - does not occur: AST or ALT more than 8 times of ULN or AST or ALT more than 5 times of ULN for more than 14 days or AST or ALT more than 3 times ULN and (T Bili more than 2 times of INR or ULN more than 1.5 or AST or ALT more than ULN with the manifestation of vomiting, nausea, fatigue, right upper quadrant tenderness or pain, rash, fever, and/or eosinophilia more than 5%), it is recommended to keep up taking the drug with continuous monitoring of liver function and plasma concentration. If any of the criteria for discontinuing azoles occurs in accordance with FDA guidelines, it is advised to discontinue the administration of azoles. If the criteria are not present, it is recommended to continue taking the medications. Then, a decision should be made to change the treatment regimen containing azoles after the liver damage has resolved or improved.

For many patients with immunodeficiency disorders and those under intensive care, receiving antifungal medications is critical. However, more research is needed to evaluate the extent of their hepatotoxicity and to select appropriate drugs according to cost-effectiveness and the side effects profiles.
